# Kinetics of immunoglobulins in septic shock patients

**DOI:** 10.1186/cc9689

**Published:** 2011-03-11

**Authors:** C Siqueira, C David, C David

**Affiliations:** 1 University Hospital, Rio de Janeiro, Brazil

## Introduction

The mechanisms of sepsis are not understood in all aspects. We decided to measure the IgG and IgM serum level in these patients and tried to correlate our results with the mortality rate and also to establish the medium time in the blood of these immunoglobulins.

## Methods

We selected patients according to the Bonne and colleagues classification of septic shock. As soon as the patients were selected we took samples at entrance, day 1, day 4 and day 8. We measured the serum level of IgG and IgM of all patients. There were 189 patients studied from 360 with septic shock. We excluded 171 patients for three reasons: they were neutropenic, had transfusions for <1 month or had recently undergone chemotherapy. Septic patients represented 17% of all patients in the ICU.

## Results

From these 189 selected patients we had a mortality rate of 59 patients, which means 31%. From these patients 29 had combined deficiency of IgG and IgM levels, 17 had only IgG deficiency and 13 had IgM deficiency. We considered a deficient value as levels less than the minimum level for immunoglobulins according to our nephelometry measurement.

## Conclusions

Despite the fact that we had a small number of patients we can conclude that these measurements could be considered good prognostic markers, not only in terms of mortality rate but also to demonstrate that IgG and IgM levels do not have the 21 and 7 days of medium time in the circulation we can see in normal patients. Probably in the near future we could include immunoglobulin determination on a routine basis for septic shock patients.
